# Performance comparison of micro-neutralization assays based on surrogate SARS-CoV-2 and WT SARS-CoV-2 in assessing virus-neutralizing capacity of anti-SARS-CoV-2 antibodies

**DOI:** 10.1099/acmi.0.000257

**Published:** 2021-08-26

**Authors:** Inna Sekirov, Martin Petric, Erin Carruthers, David Lawrence, Tamara Pidduck, Jesse Kustra, Jonathan Laley, Min-Kuang Lee, Navdeep Chahil, Annie Mak, Paul N. Levett, Emelissa Mendoza, Heidi Wood, Mike Drebot, Mel Krajden, Muhammad Morshed

**Affiliations:** ^1^​ British Columbia Centre for Disease Control Public Health Laboratory, Vancouver, British Columbia, Canada; ^2^​ Department of Pathology and Laboratory Medicine, University of British Columbia, Vancouver, British Columbia, Canada; ^3^​ National Microbiology Laboratory, Winnipeg, Manitoba, Canada

**Keywords:** SARS CoV-2, COVID-19, PRNT, enzyme immunoassay, microneutralization, neutralization, serology

## Abstract

We compared neutralization assays using either the wild-type severe acute respiratory syndrome coronavirus 2 (SARS-CoV-2) virus or surrogate neutralization markers, using characterized sera. We found the results of the neutralization assays 75 % concordant overall and 80 % concordant for samples with high antibody levels. This demonstrates that commercial surrogate SARS-CoV-2 assays offer the potential to assess anti-SARS-CoV-2 antibodies’ neutralizing capacity outside CL-3 laboratory containment.

## Introduction

Nucleic acid testing is the standard for diagnosing acute severe acute respiratory syndrome coronavirus 2 (SARS-CoV-2) infection [1]. However, serological tests are instrumental in determining other epidemic factors such as community prevalence and asymptomatic infection rates [2]. Many commercially available highly sensitive and specific SARS-CoV-2 serological tests have been developed. Most of these detect antibodies that bind different structural epitopes, but cannot indicate the antibody’s virus-neutralizing capacity, namely the inhibition of virus replication by the patient serum. The gold standard for this is the plaque reduction neutralization test (PRNT), a lengthy multi-step test performed using live virus [3]. During the severe acute respiratory syndrome coronavirus 1 (SARS-CoV-1) outbreak, an alternative micro-neutralization antibody assay was implemented [4], measuring antibody-mediated inhibition of viral cytopathic effect in tissue culture. The micro-neutralization method has also been successfully used to test the neutralizing capacity of anti-H1N1 serum [5]. The micro-neutralization assay can also be applied to determine the SARS-CoV-2-neutralizing antibody levels of convalescent sera. However, SARS-CoV-2 is an RG3 pathogen, and so tests such as PRNT and micro-neutralization can only be performed in appropriate containment (CL-3) laboratory facilities. An alternative approach to the use of live SARS-CoV-2 is to produce pseudoviruses in VSV or lentivirus systems that carry the spike protein of SARS-CoV-2. Pseudovirus neutralization can be shown via the micro-neutralization method and can be performed under CL-2 containment, but still requires cell culture. In this report we describe the results of testing using a cell culture-independent commercial ELISA assay, designated as the surrogate virus neutralization test (sVNT), which measures the serum-induced inhibition of SARS-CoV-2 spike protein receptor binding domain (RBD) attachment to the host cell receptor, angiotensin converting enzyme-2 (ACE-2).

## Methods

### Samples

Two panels of serum samples were studied. A serum panel from the National Microbiology Laboratory (NML) included sera from 21 PCR-confirmed SARS-CoV-2 patients and 19 sera from patients who were not infected with SARS-CoV-2. A BCCDC PHL serum panel comprised 42 sera from PCR-confirmed and sero-positive COVID-19 patients, and 49 sera collected before the onset of the coronavirus disease 2019 (COVID-19) pandemic. All sera from COVID-19 PCR-confirmed patients had been shown to be reactive for anti-SARS-CoV-2 antibodies by at least two commercial assays that detected anti-spike antibodies: Siemens ADVIA Centaur SARS-CoV-2 Total (S1 RBD antigen) and Ortho Vitros SARS-CoV-2 total antibody (RBD antigen). Further, 39/42 samples were also reactive by a third commercial assay that detected anti-nucleocapsid antibodies (Abbott SARS-CoV-2 IgG assay). Samples that were reactive on commercial platforms were selected to represent a range of signal to cut-off (S/CO) ratios.

### Conventional virus microneutralization test (cVNT)

The challenge virus used in these assays was obtained from the NML, and was designated as hCoV-19/Canada/ON-VIDO-01–2020. The cVNT was carried out as follows: twofold dilutions of sera were heated at 56 °C for 30 min to inactivate complement. To each dilution, 100 TCID_50_ of SARS-CoV-2 was added, incubated for 2 h at 37 °C, and then transferred to microtitre plate wells containing monolayers of Vero E6 cells. Each sample was tested in duplicate. The development of cytopathic effect (CPE) was monitored over 3 days; the neutralizing titre was the lowest dilution without CPE. If only one well at the 1 : 8 dilution showed CPE, the CVNT result was recorded as equivocal.

### Plaque reduction neutralization test (PRNT)

The SARS-CoV-2 PRNT was performed as described elsewhere [6].

The highest serum dilutions resulting in 50 and 90% reduction in plaques compared with controls were defined as the PRNT_50_ and PRNT_90_ endpoint titres, respectively. PRNT_50_ titres and PRNT_90_ titres ≥1 : 20 were considered positive for SARS-CoV-2-neutralizing antibodies.

### Surrogate virus microneutralization test (sVNT)

sVNT tests (GenScript USA, Inc., NJ, USA) were performed following the manufacturer’s instructions. Diluted sera (1 : 9 dilution) were mixed with HRP-conjugated SARS-CoV-2 RBD at 1 : 1 ratio and incubated at 37 °C for 30 min. One hundred microlitres of the mixture was added to a microtitre plate and incubated at 37 °C for 15 min. The plate was then washed four times with wash buffer, before the addition of 100 µl 3,3′,5,5′-tetra-methylbenzidine solution per well, followed by incubation in the dark at 20–25 °C for 15 min. Stop solution was added to each well and the plate was read immediately at 450 nm. Results were considered to be positive for neutralizing antibodies if the percentage inhibition of the sample was 20 % or greater.

## Results

For the NML panel, PRNT_50_/PRNT_90_, cVNT and sVNT assays yielded comparable results for all 40 samples (Table 1). Of 19 SARS-CoV-2-negative samples, 1 sample had detectable neutralizing capacity by sVNT but not PRNT_50_/PRNT_90_ and cVNT assays. This sample was obtained from a recovered SARS-CoV-1 patient and the cross-reaction is not entirely surprising, considering the ~73 % similarity of the RBD motif of the two viruses. The only other SARS-CoV-1-positive sample on the panel was negative by all three tested assays. Of 21 samples from SARS-CoV-2-positive patients, 20 had fully concordant positive results by all assays and 1 had a positive sVNT and PRNT_50_ result but negative cVNT and PRNT_90_ results, potentially indicative of the ability of sVNT assay to detect low(er) levels of neutralizing antibodies (Table 1).

**Table 1.** Comparison of PRNT_50_/PRNT_90,_ cVNT and sVNT results for a well-characterized panel of samples*

		PRNT_50_		PRNT_90_		cVNT	
		POS	NEG	POS	NEG	POS	NEG
True POS (*n*=21)	sVNT POS	21	0	20	1	20	1
	sVNT NEG	0	0	0	0	0	0
True NEG (*n*=19)	sVNT POS	0	1†	0	1†	0	1†
	sVNT NEG	0	18	0	18	0	18

*Characterized samples were obtained from the National Microbiology Laboratory, Winnipeg, Manitoba, Canada. PRNT, plaque reduction neutralization assay; PRNT_50_, 50 % reduction of plaque compared to control; PRNT_90_, 50 % reduction of plaque compared to control; cVNT, conventional viral neutralization test; sVNT, surrogate viral neutralization test.

†SARS-CoV-1-positive/SARS-CoV-2-negative sample.

**Fig. 1.**
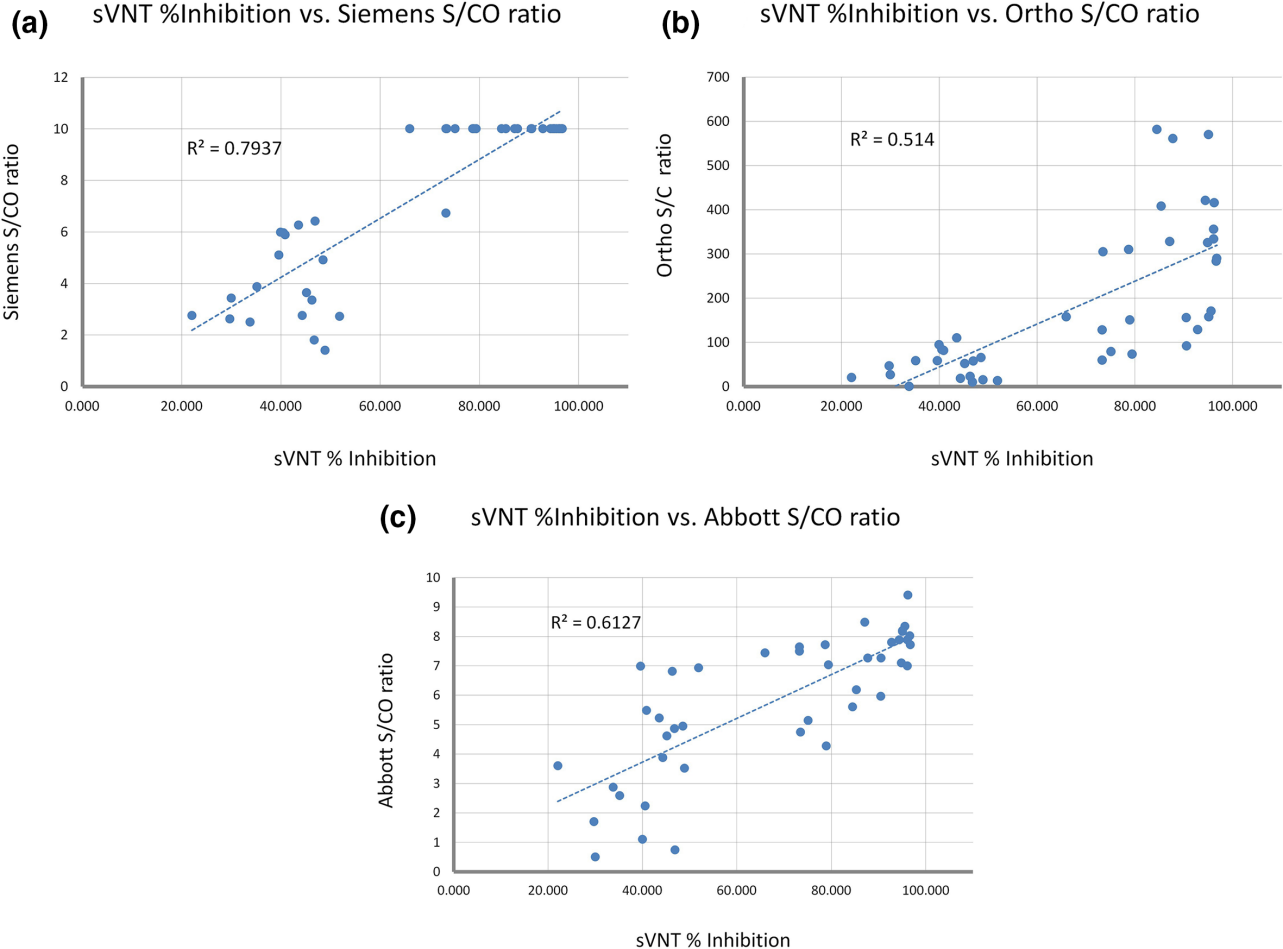
Percentage binding inhibition by sVNT assay relative to S/CO ratios obtained for positive samples on Siemens (**a**), Ortho (**b**) and Abbott (**c**) high volume CLIA serology platforms. S/CO, signal-to-cutoff ratio; sVNT, surrogate viral neutralization test.

All BCCDC PHL samples negative for SARS-CoV-2 antibodies by high-volume platforms (Siemens, Ortho and Abbott) were also negative on the sVNT assay and had titres of <1 : 8 on cVNT (Table 2). All negative samples showed inhibition of binding of less than 11 % in the sVNT. All samples that tested positive by commercial serology assays (*n*=42) were positive for inhibition by sVNT, but cVNT results were variable (Table 2). For samples testing positive in both cVNT and sVNT assays, the extent of antibody-mediated inhibition of binding to ACE-2 did not correlate with time of sample collection from disease onset, with variable levels of inhibition observed at both periods. The level of inhibition in sVNT showed a higher correlation with indices generated in the Siemens assay (Fig. 1a) than with the S/CO ratios of either the Abbott and Ortho assays (Fig. 1b, c). Samples from known positive patients with negative cVNT titres (<1 : 8) showed lower inhibition by sVNT compared to samples positive for neutralizing antibodies by both assays (44 vs 92 %). For the BCCDC panel, there was an overall positive agreement of 62 % between the sVNT and cVNT assays for COVID-19-positive sera and 100 % negative agreement on pre-pandemic sera; positive agreement increased to 71 % when equivocal results by cVNT were counted as positive.

**Table 2.** Comparison of sVNT and cVNT results for a BCCDC PHL panel of serum samples from SARS-CoV-2-infected and uninfected patients

Sample		Siemens S/CO		sVNT result		cVNT result	
Time collected	(*n*)	Result	(*n*)	Result	(*n*)	Result	(*n*)
Pre-pandemic	49			NEG	49	NEG	49
0–13 days	11	1–4.9	2	POS	2	NEG	2
from disease onset		5->10	9	POS	9	POS	9
	31	1–4.9	9	POS	9	NEG	8
≥14 days						POS	1
from disease onset		5->10	22	POS	22	NEG	2
						EQ	4
						POS	16

sVNT, surrogate viral neutralization test; cVNT, conventional viral neutralization test.

## Discussion

We compared the GenScript sVNT assay to cVNT, PRNT and commercial CLIA assays. There was a high concordance of results for PRNT, cVNT and sVNT assays on the panel received from the NML, while the BCCDC panel yielded more discrepant results by sVNT and cVNT. Overall, sVNT–cVNT agreement on sera from patients diagnosed by PCR for the combined NML–BCCDC panels was 75 % (or 81 % with equivocal cVNT results included as positive). Detectable inhibition of virus/viral epitopes binding to ACE2 receptor by sVNT but not cVNT assay could be indicative of the higher sensitivity of the sVNT method. For a single positive NML sample sVNT demonstrated inhibition concordant with PRNT_50_ results, while PRNT_90_ and cVNT results were negative. sVNT–cVNT concordance was greater for samples with higher S/CO ratios in commercial assays, but test outcome was unaffected by time of collection from disease onset. Similar findings were reported recently using two sVNT assays [7]. This is in contrast to recently reported results for GenScript sVNT, wherein samples collected >14 days post-disease onset were more likely to be positive by sVNT than samples collected <14 days after onset [8]. Moreover, while Meyer *et al*. [8] found a pseudovirus-based VNT assay to be more sensitive than the GenScript sVNT assay, we found that the GenScript sVNT assay, at least in our hands, was more sensitive than a SARS-CoV-2-based cVNT assay. This could be due to the different *in vitro* infectivity of SARS-CoV-2 vs the VSV-based pseudovirus for host cells, as well as the variability of epitopes present for neutralization on SARS-CoV-2 virus, vs those utilized in the production of pseudovirus constructs, vs those utilized in the production of competitive inhibition surrogate neutralization assays. Given that during the natural infection, the host immune system is expected to generate antibodies against an array of exposed epitopes, partial availability of epitopes for neutralization/inhibition may contribute to differences in results between cVNT, sVNT and pseudovirus VNT assays. This, similarly, can also play into the discrepancies with regard to the presence/absence of observed differences in neutralizing capacity of sera collected early/later post-infection. Depending on whether the epitope of choice for the surrogate or pseudovirus system is the main target of generated antibodies both early and later in infection, the expected results would differ. Such considerations might become particularly important when assessing cross-neutralizing capacity post-vaccination vs post-infection with different variant strains of SARS-CoV-2.

If sVNT is indeed able to detect lower titres of neutralizing antibodies, it is yet to be determined whether these lower levels translate into *in vivo* virus-neutralizing capacity. Similarly, the potential requirement to modify ‘positive’ cut-offs on sVNT assay(s) for observed inhibitory effect needs to be evaluated. Interpretations of all these assays are further complicated by the fact that there are currently no clear *in vivo* correlates established for either the cVNT or the PRNT_50/90_ assays.

A limited number of studies have been conducted on SARS CoV-2 neutralization tests [9, 10]. It has been shown that neutralizing antibody titres may vary among recovered COVID-19 patients [11], which suggests that other immune markers such as T -cells and cytokines are likely to contribute to viral clearance. Recently, Prévost *et al*. [12] found the neutralizing capacity of serum to substantially decrease in hospitalized COVID-19 patients over time, which is somewhat concordant with our cVNT findings. Whether and over what period neutralizing antibody titres correlate with protection *in vivo* still needs to be determined. Laboratory-developed [13] and commercially available sVNT assays allow for accessible high-volume assessments of antibody neutralizing capacity [14]. However, where clinical applications are concerned, suitable positive and negative cut-offs, ideally against *in vivo* correlates of protection, need to be evaluated and established.
